# A Mental Odd-Even Continuum Account: Some Numbers May Be “More Odd” Than Others and Some Numbers May Be “More Even” Than Others

**DOI:** 10.3389/fpsyg.2018.01081

**Published:** 2018-06-28

**Authors:** Lia Heubner, Krzysztof Cipora, Mojtaba Soltanlou, Marie-Lene Schlenker, Katarzyna Lipowska, Silke M. Göbel, Frank Domahs, Maciej Haman, Hans-Christoph Nuerk

**Affiliations:** ^1^Department of Psychology, University of Tuebingen, Tuebingen, Germany; ^2^LEAD Graduate School and Research Network, University of Tuebingen, Tuebingen, Germany; ^3^Department of Psychology, University of Warsaw, Warsaw, Poland; ^4^Department of Psychology, University of York, York, United Kingdom; ^5^Institute for German Linguistics, University of Marburg, Marburg, Germany; ^6^Leibniz-Institut für Wissensmedien, Tuebingen, Germany

**Keywords:** parity judgment, markedness, numerical properties, prototypicality, cross-linguistic comparisons

## Abstract

Numerical categories such as parity, i.e., being odd or even, have frequently been shown to influence how particular numbers are processed. Mathematically, number parity is defined categorically. So far, cognitive, and psychological accounts have followed the mathematical definition and defined parity as a categorical psychological representation as well. In this manuscript, we wish to test the alternative account that cognitively, parity is represented in a more gradual manner such that some numbers are represented as “more odd” or “more even” than other odd or even numbers, respectively. Specifically, parity processing might be influenced by more specific properties such as whether a number is a prime, a square number, a power of 2, part of a multiplication table, divisible by 4 or by 5, and many others. We suggest that these properties can influence the psychologically represented parity of a number, making it more or less prototypical for odd- or evenness. In the present study, we tested the influence of these numerical properties in a bimanual parity judgment task with auditorily presented two-digit numbers. Additionally, we further investigated the interaction of these numerical properties with linguistic factors in three language groups (English, German, and Polish). Results show significant effects on reaction times of the congruity of parity status between decade and unit digits, even if numerical magnitude and word frequency are controlled. We also observed other effects of the above specific numerical properties, such as multiplication attributes, which facilitated or interfered with the speed of parity judgment. Based on these effects of specific numerical properties we proposed and elaborated a parity continuum account. However, our cross-lingual study also suggests that parity representation and/or access seem to depend on the linguistic properties of the respective language or education and culture. Overall, the results suggest that the “perceived” parity is not the same as objective parity, and some numbers are more prototypical exemplars of their categories.

## Introduction

Parity judgment—that is, deciding whether a number is even or odd—is one of the earliest mathematical tasks learned in school. Formally, parity can take one of two values: an even number is an integer of the form *n* = 2*k*, while an odd number is an integer of the form *n* = 2*k* + 1. Going further, in group theory^1^, even and odd numbers build a ring with a zero element (neutral element of addition, i.e., even numbers) and a 1-element (neutral element of multiplication, i.e., odd numbers).

Thus, mathematically, parity is clearly defined. The aim of the present study was, however, to explore how parity is processed cognitively. While cognitive and psychological accounts so far have followed the mathematical definition and defined parity in terms of a categorical psychological representation, the present study aimed at testing an alternative account: Cognitively, parity may be represented in a more gradual manner, such that some numbers are represented as “more odd” or “more even” than other odd or even numbers, respectively. While this may seem an irritating concept for some numerical cognition researchers at first sight, we actually borrow from old ideas, which we apply to the concept of parity. Prototype theory (e.g., Posner and Keele, 1968; Rosch et al., 1976; Osherson and Smith, 1981) has long suggested that certain members of distinct categories are more typical examples of that category than others and that membership to such a category may be graded. Using such a theoretical conceptualization, a difference between formal binary categories and graded psychological processing can even be found in number processing, namely in processing numerical magnitude: The time needed to make (binary) same-different numerical judgments depends on the difference in magnitude between numbers (Dehaene and Akhavein, 1995, see also Sasanguie et al., 2011). Similarly, the time needed for numerical comparisons increases with decreasing distance between the numbers to be compared (numerical distance effect; Moyer and Landauer, 1967). However, for parity processing, such a graded account has—to the best of our knowledge—not been systematically tested yet (but see Armstrong et al., 1983 for an early account).

### The odd-even continuum: tentative account of the influence of numerical properties on perceived parity based on prototypicality

Several studies conducted to date have suggested that participants' responses to the parity of different numbers vary. Smallest Space Analyses (SSA-I; Guttman, 1968; Lingoes and Roskam, 1973) conducted by Nuerk et al. (2004) show that zero is located further away (i.e., processed differently) from other numbers in a parity judgment task. While Nuerk et al. (2004) only suggested that the number zero is distinct, we wish to go beyond this claim here: We suggest that many more or maybe all numbers are represented differently with regard to parity on a graded, continuous dimension. Indeed, as a small side claim in their seminal SNARC article, Dehaene et al. (1993) proposed that the mental representation of parity is influenced by several semantic properties, and pointed out that some numbers might be more prototypically odd or even. By extending this claim, one might hypothesize that specific properties facilitate or impede number processing, implying further that numbers are represented on an “oddness” or “evenness” continuum.

Dehaene et al. (1993) propose that prototypical numbers (i.e., numbers sharing many of the properties contributing to perceived parity) are classified faster as odd or even. One can postulate that one of the main factors contributing to perceived oddness and evenness would be the subjective ease of divisibility, as the parity concept itself strictly refers to divisibility by 2. The easier the division of a given number, the less subjectively odd/more subjectively even the number should be. This assumption meshes well with research on prototypicality (e.g., Rosch, 1975; Rosch and Lloyd, 1978) showing that some objects within a given category are categorized faster than others because they are (proto) typical exemplars of that category. To illustrate the point, among single-digit even numbers, 4, and 8 are powers of 2, potentially making them especially subjectively even. Only the number 6 in this set is not a power of 2 and is not divisible by 4, and as reported by Dehaene et al. (1993), the number 6 was an outlier in a parity judgment task, invoking exceptionally long reaction times. More recent studies have found that zero (Nuerk et al., 2004), 2^2^, and 6 (reanalysis of data reported in Cipora and Nuerk, 2013) among even numbers are outliers prompting longer reaction times.

While some properties are expected to influence the perceived “evenness” of a number, other properties should influence the perceived “oddness,” For instance, whether a number is prime may contribute to its subjective oddness. Notably, numbers 1 and 9 are the only one-digit odd numbers that are not prime numbers, and a reanalysis of data reported by Cipora and Nuerk (2013) showed that in the case of odd numbers, reactions to the number 9 were the slowest among odd numbers. Dehaene et al. (1993) presented similar findings, with numbers 1 and 9 invoking longer reaction times than 3, 5 and 7.

These factors may explain the general patterns in one-digit numbers, but of course cannot be systematically tested in one-digit numbers, given that there are too few numbers and too many degrees of freedom (e.g., almost all one-digit odd numbers are also primes, almost all even one-digit numbers are also powers of two; see above). These confounds are also reflected in the inconclusive results of experiments using single-digit numbers. However, such assumptions can be tested for two-digit numbers, which we therefore set out to investigate here.

**Figure 1:**
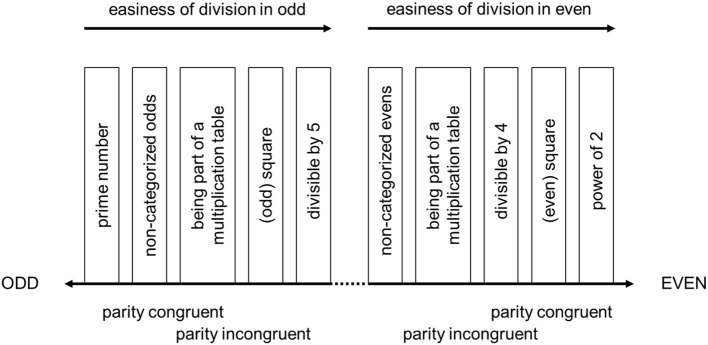
Tentative account of numerical properties and perceived parity.

We suggest the “parity continuum” as a tentative account of the influence of numerical properties on the parity representation of two-digit numbers. In line with the properties investigated by Dehaene et al. (1993), we included being a *prime number* (being divisible only by one and by itself, e.g., 23) and being a *power of 2* (e.g., 32, 64) as prototypical numerical properties for being odd and even, respectively. These two properties constitute extremes of perceived easiness of division (cf. Figure 1). Nevertheless, there are several other properties that can conceivably affect parity judgments, and which also influence the ease of division. These properties will be outlined in the following paragraphs.

With respect to easiness of division, it is easy to recognize numbers *divisible by 5* using a very simple heuristic. Furthermore, studies investigating the relationship between finger-counting habits and number processing suggest a key role of 5 as a sub-base in mental quantity representation and arithmetic. Such sub-base-5 effects have been observed in a number comparison task (Domahs et al., 2010), and a completing-addition production task (Klein et al., 2011). For these two reasons, we postulate that divisibility by 5 decreases the perceived oddness of a given number. At this stage in our tentative model we do not consider even numbers which are divisible by 5, that is full decades. In the base-10 system, decade numbers are special for many reasons (e.g., length, role in the base-10-system, e.g., for carry overs, consistency effects in multiplication and so forth; see e.g., Nuerk et al., 2002, 2015).

For even numbers, *divisibility by 4* also makes division more accessible, because the result of the division by 2 is also even (i.e., divisible by 2). This claim is supported by unpublished data collected by one of the co-authors (H-CN), which show that numbers divisible by 4 have unique characteristics compared to other even numbers. In that sense, divisibility by 4 increases perceived evenness of the number.

Following the easiness of division account, it must be noted that numbers that are *part of multiplication tables* are divisible by definition. Furthermore, in many educational systems, multiplication tables are learned by rote memorization. Therefore, we are more familiar with these numbers. They can be processed more easily than numbers we rarely encounter, which was shown in several studies. For instance, in number bisection tasks, participants tend to respond faster and more accurately to items with numbers that are part of the same multiplication table (Nuerk et al., 2002). Therefore, being part of the multiplication table decreases perceived oddness and increases the perceived evenness of the number. Furthermore, even numbers constitute the majority (75%) of results of the multiplication table (because odd × odd is the only combination leading to an odd multiplication result). In line with the easiness of division and familiarity notions, we also added being a *square number* to the account. As French (2005) points out, special attention is put on square numbers in mathematics lessons, which increases their familiarity and, akin to the other numbers that are part of a multiplication table, might influence their prototypicality and, thus, how their parity is processed. Being a square may decrease the perceived oddness and increase the perceived evenness of a number, because even numbers are probably generally more familiar. In Figure 1 we present a tentative model of the parity continuum account, in which apart from the abovementioned properties we included the postulated positions of odd and even numbers that are not characterized by any of them. The order of categories depends on the postulated easiness of division within both odd and even numbers.

Empirical studies on parity judgments in two-digit numbers indicate that more than just the mathematical properties of the number influence reaction times. Namely, participants tend to respond faster to two-digit numbers if the number's decade and unit have the same parity status (both even: e.g., 48; both odd: e.g., 73), and respond slower if the parity status of the decade and the unit differ from each other (one even, one odd: e.g., 32, 45; Dehaene et al., 1993; Tan and Dixon, 2011). This effect, referred to as *parity congruity* is one of the 17 effects suggested to indicate decomposed processing of multi-digit numbers (Nuerk et al., 2011a,b for reviews). Although it is not an attribute related to division and multiplication (and therefore not depicted in Figure 1), parity congruity influences the ease of the parity decision and needs to be taken into account.

To sum up, properties related to divisibility, sub-base and familiarity as well as parity congruity seem to influence the perceived parity of two-digit numbers. What is more, one can point to a number of linguistic factors that need to be taken into consideration while investigating numerical processing.

### Linguistic factors influencing number processing

Numerical processing is also affected by linguistic features (see e.g., Dowker and Nuerk, 2016). Previous studies show that processing (i.e., accessing and operating with) even numbers might be different from processing odd numbers. One of the effects explained by linguistic factors is the so called “odd effect”: In a traditional parity judgment task, people tend to respond faster to even numbers than to odd numbers (Hines, 1990). It is often explained by the concept of linguistic markedness. It assumes that adjectives are arranged in pairs, which contain a marked, basic form and an unmarked one—the derived one. The unmarked form is the “more natural” form of an adjective and the marked form in some cases can be even produced out of the unmarked form by adding a negation prefix. In other cases, the marked form is identified as being less frequent (e.g., we ask “How old are you?”/“How long does it take?” rather than “How young are you?”/“How short does it take?” see e.g., Nuerk et al., 2004; Huber et al., 2015; Schroeder et al., 2017). It is also relatively easy to indicate markedness of adjectives referring to number parity. Evenness is considered as the unmarked from, and oddness as a marked one. In English, the word odd apart from denoting numbers indivisible by 2, means also “weird” or “non-typical”). In German and in Polish, adjectives denoting odd numbers are built by adding negation prefixes to adjectives denoting even numbers (“*un*gerade” and “*nie*parzysty” respectively). As shown in previous studies, the unmarked adjective-forms can be retrieved faster (Sherman, 1976), possibly explaining why even (“unmarked”) numbers are responded to faster than odd (“marked”) numbers.

In the case of multi-digit numbers, another linguistic property known as the inversion property is of particular importance. German two-digit number words are inverted: The unit digit is articulated first, followed by the decade digit (e.g., 25 is “fünfundzwanzig”—“five-and-twenty”). In other languages, like English or Polish, the structures of the number word systems are comparable to the Arabic number notation, i.e., the decade digit is articulated first and followed by the unit digit. The inversion property in German can lead to problems with transcoding, i.e., children mixing up units and decades when writing numbers on dictation (Zuber et al., 2009). Transcoding in inverted number systems seems to demand more working memory and executive function resources (Imbo et al., 2014). Inversion can also affect symbolic arithmetic in German-speaking children (Göbel et al., 2014). Effects of inversion on arithmetic performance (Van Rinsveld et al., 2015) and magnitude judgments (Van Rinsveld et al., 2016) can also be observed in adults. Comparing the German number word system with the Japanese (i.e., a more transparent) number word system, German-speaking children show not only more transcoding errors in general, but a specific pattern of transcoding errors reflecting the unit-decade inversion property in their number word system (Moeller et al., 2015). Additionally, due to the inversion property, German-speaking participants automatically pay more attention to the unit digit of a given two-digit number word, as this digit is articulated first, while English speaking participants tend to pay more attention to the decade digit (Nuerk et al., 2005a). This effect is present in different modalities: In non-inverted languages, decades seem to play a greater role in processing than units, regardless of whether numbers are presented visually or auditorily (Macizo and Herrera, 2008, Exp. 3; Macizo and Herrera, 2010). This prioritizing of either the unit or decade digit might influence participants' performance in number processing tasks in which units play a decisive role. Parity judgement is clearly one of those tasks, because only the unit (parity) is relevant for answering correctly.

However, not only the composition of number words influences number processing, but also the grammatical number (singular, plural) assigned to a number (Roettger and Domahs, 2015). Most languages, like English and German, follow simple rules regarding grammatical number: While 1 is associated with singular, all other numbers are associated with plural. In Polish, grammatical number rules in verbal inflection are more complex: while 1 is associated with singular, 2, 3, and 4 are associated with plural, but 5–9 are again associated with singular. The grammatical number for multi-digit numbers follows analogous rules. All numbers ending in 1 (as well as teens and full decades) are associated with singular, all numbers ending in 2, 3, or 4 are associated with plural and all numbers ending in a number from 5–9 are associated with singular again. For example, 24 is associated with plural (“There are 24.”), and 27 is associated with singular (“There is 27.”). These grammatical number rules cause an incongruence between numerical and grammatical number for numbers associated with singular grammatical number, which could have an impact on their representation and processing. Nevertheless, such influences have not yet been demonstrated and this point needs to be treated as a rather tentative prediction.

Altogether, linguistic factors are expected to influence number processing, and, therefore, to affect response speed for parity judgment. Thus, we expect reaction times for the examined numerical properties to differ cross-linguistically. Due to these linguistic influences, our initial account might not accurately depict the effect of the odd-even continuum for different language groups.

### Other factors influencing numerical judgments: magnitude and word frequency

Numerous studies investigating numerical processing point out that numerical magnitude and frequency of a given number word in natural language affect decision times on numerical stimuli. These effects can be observed both in parity and magnitude judgments. Therefore, we consider them as potentially influencing our results, despite being irrelevant to the postulated parity continuum account.

First of all, processing of numbers is affected by their magnitude. Larger numbers are associated with longer reaction times in number comparison tasks (i.e., the size effect; Moyer and Landauer, 1967). In parity judgement tasks, the size effect has also been reported (e.g., Gevers et al., 2006), but the evidence is less conclusive (e.g., Dehaene et al., 1993; Verguts et al., 2005). Further, numerical magnitude is also mapped onto space (i.e., *Spatial Numerical Association of Response Codes*, SNARC effect). Namely, in bimanual decision tasks, reactions to small/large magnitude numbers are faster on the left/right hand side (Dehaene et al., 1993; Fias, 2001; Nuerk et al., 2005b, for auditory stimuli). For two-digit numbers, SNARC effects can be found depending on the magnitude of the whole number (Tlauka, 2002), the unit magnitude (Huber et al., 2015) and the decade magnitude (Dehaene et al., 1993). Thus, the magnitude of a whole number as well as the magnitude of the constituents of a multi-digit number have an impact on number processing. In order to control for size effects, unit magnitude and decade magnitude were taken into consideration in the present study.

Besides magnitude, the frequency of a number word (Whaley, 1978), can influence number processing. Numbers occurring more often in the natural language are responded to faster than those which are rarer (see e.g., Van Heuven et al., 2014). Nevertheless, this property is not specific to numbers, but rather reflects well-established effects observed in lexical decision tasks, that decisions on words appearing more frequently in a language are faster. To control word frequency effects, log-transformed (log_10_) frequency estimates of number words (Gielen et al., 1991) were taken into consideration.

To sum up, properties such as numerical magnitude and word frequency may play a role for numerical judgments, and thus need to be taken into account, although they are not specifically related to the parity continuum account.

### The present study

The present study aimed at testing all abovementioned numerical and linguistic factors influencing parity judgments of auditorily presented two-digit numbers within one comprehensive account.

Firstly, according to prototypicality, numbers possessing the properties included in our account (i.e., numbers appearing “more odd”/“more even”) are expected to be associated with shorter reaction times. Alternatively, according to an account based on the markedness strength, as we laid out above, odd numbers are linguistically marked and therefore slower. Linguistically, markedness is a strict category, but psychologically, its effects have been shown to be influenced by individual differences, such as handedness (e.g., Huber et al., 2015). Therefore, psychological markedness may also be a graded psychological principle, similar to parity. Still, because markedness leads to slower response times (compared to unmarked concepts), stronger markedness should lead to even slower response times. Overall, the markedness strength account predicts the opposite pattern from the prototypicality account in the case of odd numbers: That increasing oddness (i.e., stronger markedness) will be associated with longer reaction times. On the other hand, for even numbers, increasing evenness (i.e., stronger unmarkedness) according to both prototypicality and the markedness approach, should be associated with shorter reaction times (H1).

Secondly, we expected overall between-language differences in parity decisions. Namely, German speakers should show significantly shorter reaction times than the other language groups, since unit-decade inversion leads to the digit relevant for parity judgment (the unit) being pronounced first in German (H2.1). Furthermore, specific features of grammatical number in Polish and English (i.e., grammatical number incongruency in the case of more than half of the numbers in Polish), might possibly lead to slower reaction times in Polish than in English speakers, and also slower than German speakers, both due to inversion property in German and grammatical number incongruencies in Polish (H2.2).

Thirdly, linguistic properties might have specific influences on effects within the parity continuum. Effects related to properties of the decade number should be weaker in German speakers, because they can initiate the response before hearing the decade number. Therefore, they can be less affected by decade magnitude or parity congruity (H3.1). Other specific linguistic differences between the English, Polish, and German language groups are expected to influence the processing of parity (H3.2).

## Methods

### Participants

A total of 110 participants (71 female; mean age: 21.8 ± 3.9 years; range: 18–40) took part in the experiment. Out of them, 36 participants were native English speakers (23 female, mean age: 20.2 ± 2.2 years; range: 18–31), 36 were native German speakers (23 female, age: 22.2 ± 3.7 years; range: 18–33) and 38 were native Polish speakers (25 female, mean age: 23.0 ± 4.9 years; range: 18–40). All participants were right-handed and had normal or corrected to normal vision. At the time of testing none of our participants had spent more than 1 year in a foreign linguistic environment. Both parents of all participants were native speakers of the same language. None of the participants suffered from any diagnosed learning, psychiatric or neurological disorder. We obtained approval for testing from the local ethics committees at each site of data collection (York, Tuebingen, and Warsaw). Except for two Polish participants who did not specify their field of study, all participants indicated that they were university students or academic staff at the respective testing sites.

All participants gave their written consent to being tested as a participant in this experiment and were free to withdraw from participation at any point. Participants were compensated with credit points, sweets, or with monetary compensation according to local regulations at testing sites.

### Materials

The task was a bimanual computerized parity judgment task on two-digit numbers in different notations/modalities (i.e., participants were to decide whether a given number was even or odd), using the “A” (left hand) and “L” (right hand) keys on a keyboard. Response keys were labeled with colored (blue and purple) stickers. The same laptop model was used at each testing site. The task was programmed and data were collected with Presentation 18.1 software (Neurobehavioral Systems Inc., Albany California, USA).

Stimuli were the numbers from 20 to 99 (10–19 in practice sessions). Stimuli were presented as either Arabic numerals, written number words, or auditorily through the computer's speakers. Presentation modality changed after one block and the order of presentation was randomized to avoid order effects. After the first three blocks with different modalities were presented, another three blocks were presented with response-key assignment reversed.

In this article, we decided to focus on results of the auditory presentation, since linguistic effects like unit-decade inversion are expected to be most salient here. It was shown that SNARC/MARC effects can be notation/modality specific (Nuerk et al., 2004) or not (Nuerk et al., 2005b), thus, for simplicity of presentation, here we only report the modality for which we expected to observe most salient effects. Each number was presented 5 times in each block (400 trials in total). Stimuli were pseudorandomized within sets of 80 numbers. Each block was preceded by a practice session, during which accuracy feedback was given and a reminder of the correct response-key assignment was presented in the bottom line of the screen. The practice session consisted of numbers 10–19 and was repeated if an 80% accuracy threshold was not reached. Additionally, a hint card about the response-to-key assignment was placed on the left side next to the laptop and was visible for the duration of the experiment.

For the auditory presentation, each trial started with a black fixation square (25 × 25 pixels), which was presented for a random duration between 175 and 250 ms (jittered in steps of 25 ms). Subsequently, a blurred mask was presented on the screen and stimuli were presented through the speakers of the computer until a response was given or for a maximum duration of 3,000 ms. The next trial started after an inter-stimulus-interval (ISI) of 200 ms. During this time, a gray mask covered the screen. The volume of speakers was set to the maximum level, and this corresponded to the natural loudness of a person speaking next to the participant. The numbers were recorded by female native speakers of the respective languages speaking at a regular tempo. The average length of number words differed between languages: in the case of English it was 3.22 syllables, in Polish 4.94 syllables, and in German 4.11 syllables. All recordings were shorter than 1000 ms and were not adjusted to length in order to keep them natural-sounding.

### Procedure

Participants were tested individually. The order of the blocks was counterbalanced across participants. After responding to demographic questions, participants started with the parity judgment task. Both speed and accuracy were stressed in the instructions.

During a break before the change of response-key assignment and after the last block was presented, participants were asked to do paper-pencil tasks that were not further analyzed (LPS-UT3, Kreuzpointner et al., 2013; a speeded 8-mi arithmetic task, as well as AMAS, Hopko et al., 2003). A debriefing sheet was presented on request at the end of testing.

### Data preparation and analysis

#### Data exclusion

Results from practice sessions were not analyzed. The average error rate was 6.34% and errors were not analyzed due to the ceiling effect in a simple task such as parity judgement. Only reaction times associated with correct responses were further analyzed. Due to technical problems, data from three participants (one per language) were not recorded. Reaction times shorter than 200 ms were treated as anticipations and were excluded. Additionally, reaction times that deviated more than ±3 standard deviations from a participant's mean were excluded sequentially with an update of the mean and standard deviation computation after a trial was excluded until no further exclusions occurred (see e.g., Cipora and Nuerk, 2013 for the same procedure). Due to an error in the programming procedure, results of one stimulus (number 97) could not be analyzed. All these procedures resulted in another 6.46% of data exclusions, so that finally, 87.2% of the data were retained for reaction time analysis. In a second step, full decade numbers and tie numbers were discarded from the analysis as they cannot be easily compared to other two-digit numbers (Dehaene et al., 1990; Nuerk et al., 2011a, 2015), and are frequently excluded from stimuli sets (e.g., Moeller et al., 2009; Chan et al., 2011; Macizo and Herrera, 2011). Full decades are highly frequent and processed very fast (Brysbaert, 1995). For instance, bisection tasks are facilitated by including a decade number as one of three numbers in the bisectable triplet, as well as by staying in the same decade between the first and third number of the triplet (Nuerk et al., 2002; Korvorst et al., 2007; Wood et al., 2008)^3^.

#### Multiple regression analyses (H1)

Within-participant multiple regressions were calculated separately for odd and even numbers. Predictors not specifically related to the parity continuum account were included in both models. These were: (a) Log-transformed (log_10_) frequency of a number word estimated by subjective ratings, ranging from 0 to 500 (Gielen et al., 1991)^4^, (b) unit magnitude, (c) decade magnitude, (d) parity congruity. Multiple regressions for even numbers included predictors: being a *square*, being a part of a *multiplication table*, a *power of 2*, as well as being *divisible by 4*. Multiple regressions for odd numbers included predictors: being a *square*, a *prime number*, being part of a *multiplication table*, as well as being *divisible by 5*.

Binary predictors: *parity congruity*, being a *square*, a *prime number*, part of a *multiplication table*, a *power of 2*, as well as being *divisible by 4* and *by 5* were coded as 1 when the particular feature was present, and 0 when they were not. Individual regression slopes (unstandardized beta coefficients) for each predictor served as dependent measures that were further analyzed. Participants' regression slopes for each factor were tested against 0 with a two-sided *t*-test (Lorch and Myers, 1990). Levels of significance were adjusted for multiple comparisons using False Discovery Rate (FDR) correction (Benjamini and Hochberg, 1995). Positive slopes denote longer reaction times for possessing/increasing a given property; negative slopes denote shorter reaction times for possessing/increasing a given property. Regarding our prototypicality hypothesis for the effects of the odd-even continuum (H1), we expected factors which lead numbers to be processed as “more odd” or “more even” to show more negative slopes, that is, to be associated with shorter reaction times.

In order to check for predictor collinearity, we calculated correlations between predictors (See Supplementary Material A). Although in some cases correlations were moderate, they did not exceed 0.57 in any case; thus, it did not raise the problem of collinearity for multiple regressions^5^. However, to check for possible suppression effects (potentially changing the direction of relationships observed within the multiple regression approach), we calculated bivariate correlations between predictors of interest. Averaged within participant bivariate correlations are presented in Supplementary Material B. Furthermore, we checked whether slopes associated with significant effects had the same directions as averaged bivariate correlations. If that was the case, it is mentioned explicitly in the Results section. Note that the setup we used allows calculating the SNARC effect as well. Nevertheless, it was out of the scope of the present study; thus it is not presented in the following analysis, but it is reported in Supplementary Material C.

#### Group comparisons (H2.1 and H2.2; H3)

To investigate whether language groups differed in reaction times (H2.1 and H2.2) and regression slopes (H3), respectively, we calculated one-way ANOVAs. In addition, Bayesian ANOVAs were conducted. Posterior probabilities in favor of the null hypothesis model given the data *p(H*_0_*|D)* were calculated, with the null hypothesis denoting no between-group differences and the alternative hypothesis denoting between-group differences. Interpretations of posterior probabilities were based on Raftery (as cited in Masson, 2011). All analyses were conducted with R (version 3.3.0; R Core Team, 2018) and JASP (Version 0.8.2; JASP Team, 2017).

#### Comparing odd and even numbers

To investigate whether the whole sample showed an odd effect (faster mean reaction times for even than for odd numbers in general), a one-way ANOVA was calculated checking for a group difference between even and odd stimuli.

## Results

### Multiple regression analyses (H1 and H3)

#### Whole-sample level

Including all participants, multiple linear regression analysis and subsequent *t*-tests revealed significant effects in both odd and even numbers. In odd numbers, *prime number* and *divisibility by 5* showed significant, positive slopes (i.e., were associated with longer reaction times). For even numbers being a *square* and *divisibility by 4* showed negative slopes (i.e., were associated with shorter reaction times). On the contrary, being part of a *multiplication table* was associated significantly with longer reaction times in even numbers (cf. Table 1). Interestingly, the bivariate correlation with being part of a multiplication table had the opposite direction from regression slopes, suggesting the presence of suppression effects.

**Table 1:** Predictors influence on overall response times in all three languages.

	**Mean slope (SD)**	***t*_(106)_**	***p* (*q* = 0.031)**	***d***
**ODD NUMBERS**				
Decade magnitude	8.32 (7.99)	10.80	<**0.001**	1.04
Unit magnitude	4.24 (5.10)	8.60	<**0.001**	0.83
Parity congruity	−11.40 (23.30)	−5.05	<**0.001**	−0.49
Prime number	24.10 (31.70)	7.87	<**0.001**	0.76
Square	−1.03 (38.10)	−0.28	0.779	−0.03
Multiplication table	7.57 (37.30)	2.10	0.038	0.20
Divisibility by 5	27.70 (39.00)	7.35	<**0.001**	0.71
Frequency	7.66 (110.00)	0.73	0.468	0.07
**EVEN NUMBERS**				
Decade magnitude	8.29 (8.56)	10.00	<**0.001**	0.97
Unit magnitude	2.99 (8.13)	3.80	<**0.001**	0.37
Parity congruity	3.05 (25.10)	1.25	0.213	0.12
Square	−11.70 (55.40)	−2.18	**0.031**	−0.21
Multiplication table	15.40 (39.20)	4.06	<**0.001**	0.39
Power of 2	6.36 (49.40)	1.33	0.185	0.13
Divisibility by 4	−10.90 (27.10)	−4.17	<**0.001**	−0.40
Frequency	9.35 (240.00)	0.41	0.682	0.04

*q, FDR-corrected alpha level; d, Cohen's d. Significant predictors are marked with bold*.

Regarding the other predictors, *parity congruent* numbers were responded to faster than incongruent ones but only in the case of odd numbers. On the other hand, increasing *decade magnitude* and *unit magnitude* were associated with longer reaction times for both odd and even numbers. Frequency was neither significant for odd nor for even numbers (cf. Table 1). Unexpectedly, in the case of even numbers the bivariate correlation between unit magnitude and reaction times was negative, suggesting the presence of suppression effects (cf. Supplementary Material B).

#### Within-language group analyses

Subsequently, regression slopes were tested against zero separately for each language group. Checking whether given effects were observed within each language group was a necessary prerequisite for comparing language groups as a next step.

#### English

For odd numbers, *t*-tests on regression slopes revealed significant effects of being a *prime number*, being *a square*, and *divisibility by 5*. Being a prime number and divisibility by 5 were associated with longer reaction times, whereas being a square was significantly associated with shorter reaction times (cf. Table 2). In the case of even numbers, being part of a *multiplication table* was associated with longer reaction times, while being a *square* and *divisibility by 4* resulted in shorter reaction times (cf. Table 2). Notably, in the case of being part of a multiplication table, the bivariate correlation had an opposite direction suggesting the presence of suppression effects (cf. Supplementary Material B).

**Table 2:** Predictors influence on response times separately for each language.

**Odd numbers**	**English**				**German**				**Polish**			
	**Mean slope (*SD)***	***t*_(34)_**	***p* (*q* = 0.038)**	***d***	**Mean slope (SD)**	***t*_(34)_**	***p* (q = 0.013)**	***d***	**Mean slope (SD)**	***t*_(36)_**	***p* (*q* = 0.044)**	***d***
Decade magnitude	9.87 (4.50)	13.00	<**0.001**	1.25	−0.18 (5.68)	−0.19	0.851	−0.02	14.80 (4.60)	19.60	<**0.001**	1.89
Unit magnitude	1.14 (3.70)	1.82	0.078	0.18	5.68 (5.55)	6.05	<**0.001**	0.59	5.72 (4.54)	7.66	<**0.001**	0.74
Parity congruity	−17.00 (18.30)	−5.48	<**0.001**	−0.53	−5.90 (27.40)	−1.27	0.211	−0.12	−8.97 (17.70)	−3.08	**0.004**	−0.30
Prime number	27.10 (21.10)	7.58	<**0.001**	0.73	1.60 (32.70)	0.29	0.775	0.03	41.80 (26.70)	9.52	<**0.001**	0.92
Square	−13.20 (35.10)	−2.23	**0.033**	−0.22	3.46 (44.40)	0.46	0.647	0.05	6.25 (32.10)	1.18	0.245	0.11
Multiplication table	1.04 (31.20)	0.20	0.845	0.02	−15.10 (33.60)	−2.66	**0.012**	−0.26	35.90 (27.80)	7.87	<**0.001**	0.76
Divisibility by 5	38.70 (32.10)	7.13	<**0.001**	0.69	−1.12 (34.60)	−0.19	0.849	−0.02	44.10 (33.90)	7.92	<**0.001**	0.77
Frequency	71.20 (90.00)	4.69	<**0.001**	0.45	25.30 (95.00)	1.57	0.127	0.15	−66.30 (91.00)	−4.43	<**0.001**	−0.43
**Even numbers**	**Mean slope (SD)**	***t*_(34)_**	***p*** **(*****q*** = **0.031)**	***d***	**Mean slope (SD)**	***t*_(34)_**	***p*** **(*****q*** = **0.031)**	***d***	**Mean slope (SD)**	***t*_(36)_**	***p*** **(*****q*** = **0.006)**	***d***
Decade magnitude	9.26 (6.25)	8.76	<**0.001**	0.85	1.10 (7.41)	0.88	0.387	0.09	14.30 (6.40)	13.60	<**0.001**	1.32
Unit magnitude	5.13 (5.58)	5.43	<**0.001**	0.53	5.76 (9.77)	3.49	**0.001**	0.34	−2.08 (6.30)	−2.01	0.052	−0.19
Parity congruity	3.25 (26.90)	0.72	0.479	0.07	5.78 (28.60)	1.19	0.241	0.12	0.79 (20.50)	0.23	0.817	0.02
Square	−15.20 (51.50)	−1.74	0.091	−0.17	−32.80 (60.90)	−3.19	**0.003**	−0.31	9.30 (46.50)	1.22	0.231	0.12
Multiplication table	25.10 (33.80)	4.38	<**0.001**	0.42	23.00 (48.00)	2.83	**0.008**	0.27	−0.21 (29.80)	−0.04	0.966	−0.01
Power of 2	−5.34 (38.10)	−0.83	0.413	−0.08	27.80 (49.20)	3.34	**0.002**	0.32	−3.04 (53.40)	−0.35	0.731	−0.03
Divisibility by 4	−29.70 (20.30)	−8.65	<**0.001**	−0.84	1.15 (27.80)	0.25	0.807	0.02	−4.36 (22.90)	−1.16	0.254	−0.11
Frequency	212.00 (120.00)	10.00	<**0.001**	0.97	−194.00 (210.00)	−5.50	<**0.001**	−0.53	4.95 (170.00)	0.18	0.859	0.02

*q, FDR–corrected alpha level; d, Cohen's d. Significant predictors are marked with bold*.

As regards the other predictors, *parity congruent* numbers were responded to faster than incongruent ones but only in the case of odd numbers. On the other hand, increasing *decade magnitude* was associated with longer reaction times for both odd and even numbers. Increasing *unit magnitude* was significantly associated with increasing reaction times only for even numbers. Frequency was significant for both odd and even numbers (cf. Table 2). More frequent numbers were responded to slower than less frequent ones.

#### German

For odd numbers, results of *t*-tests on regression slopes revealed a significant association of being part of a *multiplication table* with shorter reaction times (cf. Table 2). In the case of even numbers, being part of a *multiplication table* or a *power of 2* were significant positive predictors, meaning possessing these numerical properties was associated with longer reaction times. In addition, being a *square* led to shorter reaction times (cf. Table 2).

As regards the other predictors, *parity congruity* and *decade magnitude* were not significant. On the other hand, increasing *unit magnitude* was significantly associated with increasing reaction times for both odd and even numbers. Frequency was significant only in even numbers (cf. Table 2). More frequent numbers were responded to faster than less frequent ones.

#### Polish

For odd numbers, being a *prime number*, being part of a *multiplication table*, and *divisibility by 5* were significant positive predictors, meaning possessing them was associated with longer reaction times. Nevertheless, the bivariate correlation between being part of a multiplication table and reaction time was negative (cf. Supplementary Material B), suggesting possible suppression effects. For even numbers, none of the specific predictors reached significance (cf. Table 2).

As regards the other predictors, *parity congruent* numbers were responded to faster but only in the case of odd numbers. Increasing *decade magnitude* was associated with longer reaction times for both odd and even numbers, while increasing *unit magnitude* was associated with longer reaction times only in odd numbers. Increasing frequency was related with shorter reaction times only in odd numbers (cf. Table 2).

### Between-group differences in mean reaction time (H2.1 and H2.2) and the odd effect

**Figure 2:**
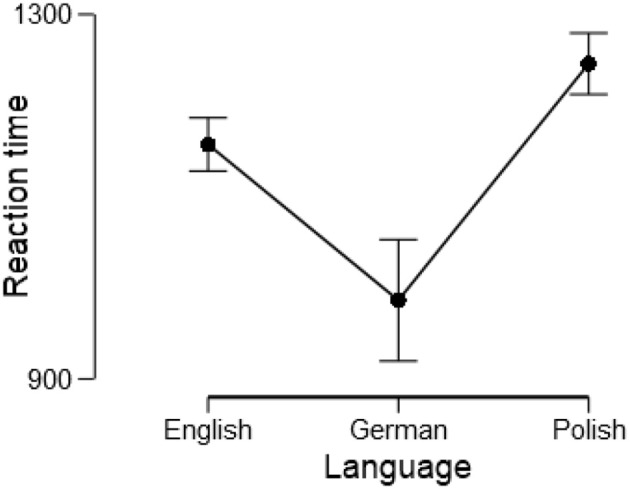
Mean reaction times with 95% confidence interval for the English, German, and Polish language group.

To address *H2.1* and *H2.2*, and to check for a presence of the odd effect, a mixed design 3 (language) × 2 (parity) ANOVA was conducted. There was a robust effect of Language, *F*_(2, 214)_ = 68.04, *p* < 0.001, $\eta_{\text{p}}^{2}$ = 0.39 (cf. Figure 2). Post hoc comparison revealed that all groups different significantly from each other (*p*s < 0.001). Interestingly, there was no main effect of number parity, *F*_(1, 214)_ = 0.24, *p* = 0.628, $\eta_{\text{p}}^{2}$ < 0.01 indicating absence of the odd effect^6^. The interaction parity × language was also not significant, *F*_(2, 214)_ = 0.02, *p* = 0.979, $\eta_{\text{p}}^{2}$ < 0.01, thus the odd effect was not modulated by language.

### Between-group comparisons (H3)

For odd numbers, ANOVAs testing for group differences in regression slopes revealed significant differences between language groups for *prime number, being part of a multiplication table*, and *divisibility by 5* (cf. Table 3). For even numbers, ANOVAs revealed significant differences between language-groups for factors *being a square, being part of a multiplication table, power of 2*, and *divisibility by 4*. To support these results, Bayesian ANOVAs were calculated, as well (cf. Table 3). As regards the other predictors, groups did not differ in *parity congruity*. On the other hand, there were differences as regards the effects of *decade magnitude, unit magnitude*, and frequency for both odd and even numbers (cf. Table 3).

**Table 3:** Predictors influence on response times as compared between three languages.

	***F*_(2, 104)_**	***p***	**$\eta_{\text{p}}^{2}$**	***p*(H0|D)**	**BF01**	***p*(H1|D)**	**Interpretation**	***Post-hoc***
**ODD NUMBERS**								
Decade magnitude	86.41	<**0.001**	0.62	0.000	0.000	1.000	Very strong for H1	All groups differ
Unit magnitude	11.56	<**0.001**	0.18	0.001	0.001	0.999	Very strong for H1	E differs from G and P
Parity congruity	2008.00	0.139	0.04	1.000	2276.000	0.000	Very strong for H0	Not applicable
Prime number	21.13	<**0.001**	0.29	0.000	0.000	1.000	Very strong for H1	All groups differ
Square	2799.00	0.065	0.05	0.999	1201.000	0.001	Very strong for H0	Not applicable
Multiplication table	24.96	<**0.001**	0.32	0.000	0.000	1.000	Very strong for H1	P differs from G and E
Divisibility by 5	19.46	<**0.001**	0.27	0.000	0.000	1.000	Very strong for H1	G differs from E and P
Frequency	21.73	<**0.001**	0.30	0.000	0.000	1.000	Very strong for H1	P differs from G and E
**EVEN NUMBERS**								
Decade magnitude	35.03	<**0.001**	0.40	0.000	0.000	1.000	Very strong for H1	All groups differ
Unit magnitude	11.04	<**0.001**	0.18	0.002	0.002	0.998	Very Strong For H1	P differs from G and E
Parity congruity	0.43	0.652	0.01	1.000	8113.000	0.000	Very strong for H0	not applicable
Square	6506.00	**0.002**	0.11	0.059	0.063	0.941	Positive for H1	G differs from E and P
Multiplication table	5363.00	**0.006**	0.09	0.133	0.154	0.867	Positive for H1	P differs from G and E
Power of 2	5321.00	**0.006**	0.09	0.139	0.162	0.861	Positive for H1	G differs from E and P
Divisibility by 4	16.60	<**0.001**	0.24	0.000	0.000	1.000	Very strong for H1	E differs from G and P
Frequency	50.33	<**0.001**	0.49	0.000	0.000	1.000	Very strong for H1	All groups differ

*H0, null hypothesis or no between-group difference, H1, alternative hypothesis or group difference, E, English, P, Polish, G, German. Significant predictors are marked with bold*.

## Discussion

Results of a parity judgment task with two-digit numbers in three language groups (English, German, and Polish) were analyzed regarding numerical properties for odd and even numbers in order to verify the parity continuum account and language differences in parity processing. We observed robust language differences in overall reaction times thus confirming hypotheses H2.1 and H2.2. Hypotheses regarding direction of mean slopes (H1), as well as linguistic differences regarding mean slopes (H3) could partially be confirmed and were partially contradicted, which will be discussed below. It was not straightforward to test the tentative account directly, because the postulated categories are neither fully independent of each other nor fully nested (e.g., odd squares are neither a subset of numbers divisible by 5, nor is it the other way around). Instead, after controlling for the effects of *parity congruity, unit*, and *decade magnitude*, as well as *frequency*, we compared the regression slopes for numerical properties potentially influencing the perceived parity with the parity continuum account. Those numerical properties comprised being a *prime number*, a *square*, part of a *multiplication table*, and being *divisible by 5* for odd numbers, as well as being a *square*, part of a *multiplication table*, a *power of 2*, and being *divisible by 4* for even numbers.

### Conclusions for the tentative account

The fundamental assumption that time needed for parity judgments differs considerably depending on numerical properties was confirmed by the data. However, the strict order postulated by neither by the prototypicality nor the markedness strength account was not fully captured.

For odd numbers, being a *prime* number and being *divisible by 5* was related to systematically longer reaction times. Despite having a robust impact on reaction times, the pattern of results was not in line with the prototypicality account's predictions that increasing easiness of division would make numbers subjectively less odd and thus associated with longer reaction times. Accordingly, primes would be responded to fastest, and numbers divisible by 5, slowest. The results were also not in line with predictions driven from the markedness strength account that “most odd” numbers, i.e., the primes would be responded to slowest.

**Figure 3:**
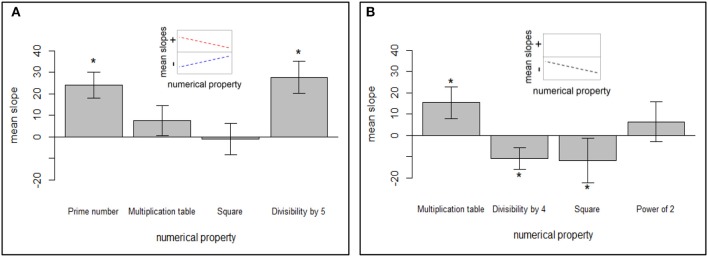
Mean slopes with 95% confidence intervals for numerical properties of **(A)** odd and **(B)** even numbers across groups; *indicating significance after correcting for multiple comparisons. Small panels represent predictions regarding the overall tendency we expected to observe. For odd numbers, according to the prediction derived from the prototypicality account, the bars in this figure should be arranged in an increasing order (schematically represented by blue line in the small panel). In case of prediction driven from the markedness strength account, the tendency is the opposite—bars should represent a decreasing order (as schematically depicted by red line in the small panel). For even numbers, there was only one prediction driven by the prototypicality account: decreasing order of bars (as schematically depicted in the small panel).

This surprising result suggests that different factors might play a role in parity decisions and thus the account considering one dimension only (i.e., easiness of division) seems too simple to explain all numerical influences. Being *part of a multiplication table* and *being a square* were not significant predictors of reaction times (cf. Figure 3) in the whole sample analyses.

In the case of even numbers, being *part of a multiplication table, divisibility by 4*, and *being a square* significantly predicted reaction times. Expectedly, divisibility by 4 and being a square were associated with shorter reaction times. This can be due to the easiness of division dimension we introduced. However, *being part of a multiplication table* was associated with longer reaction times. This surprising result needs further investigation in future studies, as numbers that are part of a multiplication table are used more often than those which are not. On the other side, being part of a multiplication table does not determine a number's parity status, and possibly, accessibility of respective division facts might be harmful for parity processing, so that one needs to verify whether division facts are specifically related to divisibility by 2. Notably, the direction of the slope was different than the direction of bivariate correlation, thus suggesting the presence of suppression effects in the case of this predictor. This should also be addressed in future studies. The effect of *being a power of 2* was not significant. Nevertheless, slopes related to being a power of 2 were estimated based on two numbers only (32 and 64), so it may be that if one would use more repetitions of these numbers in a more specific setup, it would be possible to observe a more consistent effect. Despite the suboptimal design for investigating the effect of being a power of 2, we decided to retain this predictor in our model, because we had strong predictions regarding these numbers, and we thought that excluding it could potentially decrease the overall model fit.

### Linguistic effects as limitations and refinements for the tentative account (H2.1 and H2.2; H3)

Our hypotheses regarding differences in mean overall reaction time between language groups were confirmed: German speaking participants reacted the fastest, while Polish speaking participants the slowest (H2.1 and H2.2). In the case of German participants, reaction times were shortest mostly due to the inversion property—the decisive unit number was heard first so that participants could start to give the response, or at least prepare it. This effect was indeed observed and reaction times were the fastest in German participants, despite the considerably larger syllable length of number words in German than in English. On the other hand, Polish speakers were the slowest, which might be either due to the fact that Polish number words were longest, or due to specific grammatical number properties. Note that the point in time at which specific number words are recognized differs across languages. For instance, to accurately categorize the number 91 in Polish, the decisive syllable “je,” being the first syllable of the number of units, appears in the fifth position of the number word “dziewiećdziesiat jeden,” whereas in German the decisive “ein” syllable appears in the first position of the number word “einundneunzig.”

**Figure 4:**
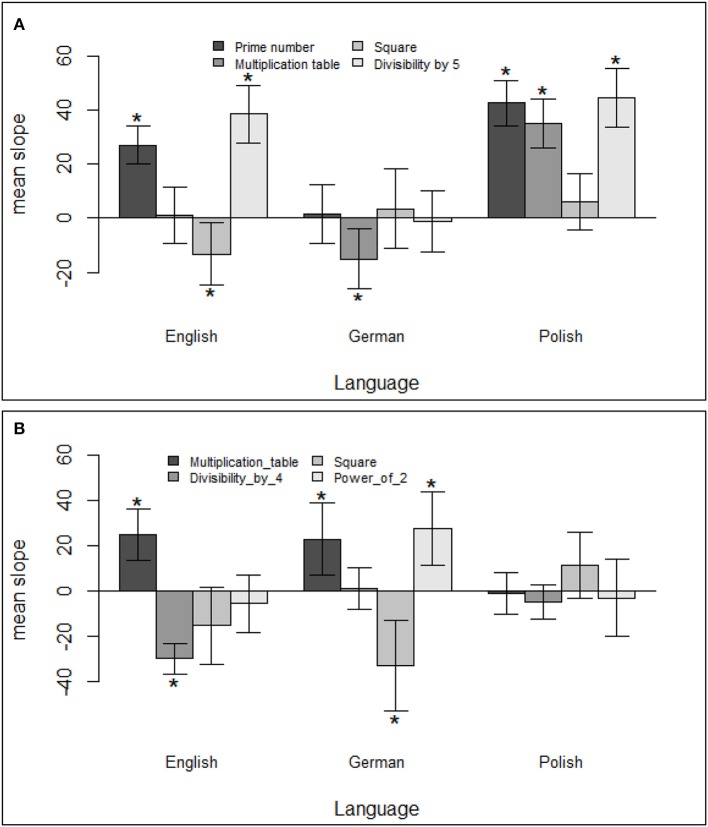
Mean slopes with 95% confidence intervals for numerical properties of **(A)** odd and **(B)** even numbers in the English, German, and Polish group; *indicating significance after correcting for multiple comparisons.

Furthermore, due to the inversion property, one might also expect that numerical properties will affect German speakers to a lesser extent than English and Polish speakers. Interestingly, this was true only in the case of odd numbers. In the case of even numbers, German speakers were highly affected by numerical properties, but Polish speakers were not (cf. Figure 4).

The overall effects of being *a prime number* and *divisibility by 5* were driven only by English and Polish speakers but were not present in German speakers. Recognizing whether a given number is a prime requires processing a whole two-digit number. Thus, the absence of an effect in German can be explained by the fact that German speakers make their parity decisions based on units only and can simply ignore the following decade number. However, the lack of an effect of *divisibility by 5* in German is puzzling. Divisibility by 5 can be accessed based on the number of units; thus, its effect should be present in German speakers as well.

Interestingly, for odd numbers being *part of a multiplication table* was a significant predictor in German and Polish speakers. Nevertheless, the direction of the effect was opposite (shorter reaction times in German and slower in Polish), and the effects canceled each other out. This means that the prototype hypothesis was corroborated in Polish. Being part of a multiplication table makes a number less typically odd (than for instance being a prime number) and therefore, RTs are slower. In contrast, for German speakers, the markedness hypothesis seems to be true in that these “less odd” numbers are faster, because they are less marked. We did not hypothesize this result. Two explanations are possible. First, maybe markedness is particularly pronounced in German, possibly because marked adjectives are often obvious, because negating prefixes are particularly common. The second hypothesis refers to multiplication learning. Possibly, learning multiplication tables is not so highly overlearnt anymore (our personal anecdotal impression from many studies is that many elementary school teachers do not like the drill associated with it) and therefore the effect of prototypicality is less pronounced than in Poland. This needs to be tested in future cross-cultural studies in which the ease of multiplication table activation is also assessed in the same participants. Another important difference as regards the prototypicality account refers to the inversion effect in German. Because the unit is spoken first, the whole multiplication number does not need to be processed before the parity decision is initiated (when one hears “seven-and-twenty,” he or she can initiate the response when he or she hears “seven”). Therefore, the activation of the identity of the whole number may be less or later. Consequently, the influence of prototypicality as derived from multiplication attributes of the whole number may be weaker in German.

English, in which no effects were found, may be a mix between Poland and Germany as regards markedness and prototypicality effects. However, we wish to note that the direction of the effect in Polish might be due to suppression. Finally, the effect of *being square* was significant only in English speakers. Since this refers to 4 numbers only (9, 25, 49, 81), we would not wish to make any strong claims at this first study on the subject.

In the case of even numbers, none of the numerical predictors reached significance in Polish speakers. In the case of English and German speakers, the effect of being *part of a multiplication table* was significant and went in the same direction (but suggests suppression effects in German). On the other hand, it seems that the overall effect of *divisibility by 4* was driven by English speakers only, while the overall effect of being *a square* was driven by German speakers only. The effects in English and German can be explained by both markedness and prototpicality as outlined above. The null effects in Polish come as a surprise but could be due to a weaker role of markedness in the Polish language that could already partially explain the effects for odd numbers. Again, this explanation is tentative and requires further specialization.

Overall, while some language effects pointed in the hypothesized direction, others were pointing in the opposite direction. Possible causes are linguistic, educational, and cultural differences, different saliencies of the prototype and markedness strength hypotheses in different languages, but also methodological issues like a small number of stimuli in some categories and possible collinearities.

To begin with, in the introduction, we outlined the prototype and the markedness strength hypotheses. For even numbers, these hypotheses predicted the same things. Multiplication attributes should lead to faster RT. For odd numbers, they predicted opposite patterns. While the prototype account predicted faster RTs for more prototypical odd numbers (e.g., prime numbers), the markedness strength account predicted longer RTs for such numbers, because they are psychologically more marked and therefore processed even slower.

The predictions for even numbers (*divisibility by 4, being a square number*) followed the prototype and markedness hypotheses. Only being part of a multiplication table was not in the expected direction. It is conceivable that this effect is due to complex suppression effects, because divisibility by 4 and being a square number overlap with multiplication effects. This tentative explanation seems to be supported by observation that bivariate correlations went in the opposite direction than multiple regression slopes.

The predictions for odd numbers are more complicated than we had anticipated. Some of the results seem to favor the prototype hypothesis, while other seem to favor the markedness strength hypothesis. Our presumption is that both hypotheses may be valid and that their saliency depends on linguistic, educational and cultural properties. For instance, being a *prime number* prolonged RT in English and Polish, thus favoring a markedness strength account for this attribute. However, it did not prolong RT in German, probably because the parity decision in German could be finished before the whole number (and hence the identity of the prime number) was finished. Similarly, the effect of *being part of a multiplication table* went in opposite directions in German and Polish. While the faster RTs in German seemed to favor a markedness strength account for this attribute, the slower RTs in Polish seemed to favor the prototypicality account. However, markedness saliency induced by parity is similar in both languages, because odd is a negation of even in both languages (“ungerade” vs. “gerade” in German, “nieparzysty” vs “parzysty” in Polish). Therefore, there might be other linguistic or cultural or educational factors, which may favor the markedness strength account in German and the prototypicality account in Polish, which we do not yet fully understand. All in all, while some patterns observed with regard to the odd numbers like the different effects of prime numbers can be explained based on the available accounts, other differences, such as multiplication table influences cannot be easily explained. We wish to acknowledge, however that because of collinearities and nested effects (prime numbers are by definition not part of the multiplication tables), suppression effects and therefore a methodological explanation rather than a theoretical remains possible.

#### Effects of congruity, size, and frequency

The factor *parity congruity* was included to investigate unit-decade congruity effects in even and odd numbers. For odd numbers, participants responded slower to incongruent stimuli at the whole-sample level, as well as in the Polish and English group, but not in the German group. This fits with the inversion property of German language, because it is easier for German speakers to ignore the task-irrelevant decade number being presented as second. For English and Polish the interfering decade number is spoken first before the response-relevant unit digit, while for German the response-relevant unit digit is spoken first and the answer can in principle be initiated before the decade digit is even presented. Interestingly, for even numbers, parity congruity did not influence reaction times, either on the whole-sample level, or in any of the three individual language groups. An explanation for this unexpected effect is tentative. However, we need to keep in mind that responding to even numbers is faster (*odd effect*, Hines, 1990). Evenness is the unmarked pole of the parity representation and is as such the more dominant ground form, which is easier to access and more salient. It is conceivable that there is an equivalent to the global precedence in global-local research (Navon, 1977; but see Kimchi, 1992) in that there is a precedence for processing even numbers, which receive less interference by odd numbers than vice versa (at least for auditory numbers and with a balanced stimuli set such as we used).

*Decade* and *unit magnitude* affected reaction times significantly for both odd and even numbers at the whole-sample level. Increasing magnitude was associated with longer reaction times. This size effect (Moyer and Landauer, 1967)—the bigger the number, the slower the response—differed significantly between language groups in both odd and even numbers.

The decade magnitude effect was present in both odd and even numbers at the whole sample level as well as in English and Polish, but not in German speakers. Again, this might be due to the inversion property of German.

The results regarding the unit magnitude are also fairly straightforward. It was apparent for both odd and even numbers at the whole sample level. Interestingly, it was present in German speakers for both odd and even numbers, which shows that magnitude effects are present in this language group but are further modulated by linguistic properties for both unit and decade digits in the expected direction. Nevertheless, the effect of unit magnitude was not present for odd numbers in English speakers or even numbers in Polish speakers. Again, the processing of unit magnitude begins later in English and Polish (because there is no inversion) and it might be weaker for the less salient odd numbers than for the more salient even numbers. In sum, the findings for decade and unit magnitude effects for different languages and for different parities largely mimic those observed for the parity congruity effect. Generally, the influence of the unit is larger in German (because of inversion), while the influence of the decade is larger in English and Polish. If there are further differences between parities, magnitude is more likely activated for even parities than for odd parities.

The *frequency* of number words was controlled for by including it as a factor in the analysis. For both odd and even numbers, *frequency* was not significant on a whole-sample level. Nevertheless, the effect of frequency was robust in both odd and even numbers in English; however, surprisingly larger frequency was associated with longer reaction times.

In the case of odd numbers in Polish and even numbers in German the effect was in line with predictions, so that higher frequency was associated with shorter reaction times. The effect was not present for odd numbers in German or even numbers in Polish. At the current stage, we do not have an explanation for this interaction between language and parity with regard to frequency effects.

## General conclusions

To begin with the hypotheses regarding, linguistic differences were robustly reflected in our results. First of all, German speakers were less affected by decade magnitude than English and Polish speakers. However, the effect of decade magnitude was not totally eliminated in this group. Namely, this group revealed some effects which depended on decade magnitude, such as responding faster to odd numbers that were part of a multiplication table. Such effects can be only explained by the decade number being at least partly processed, because such information can be extracted only when overall numerical magnitude is processed. On the other hand, inconsistent grammatical number did not play a robust role in parity decisions in Polish speakers. This might be due to the fact that numerical processing was not framed in any linguistic context in the present experiment—participants were presented with numbers only, not embedded in any additional phrasing.

Effects of multiplicativity and other numerical variables on parity could be observed but were not always consistent. For even numbers, being a *square* and *divisibility by 4* led to shorter reaction times, i.e., made a number “more even.” To give an example: 64 (square and divisible by 4) is “more even” than 62 (not a square and not divisible by 4). Note, being part of a *multiplication table* was associated significantly with longer reaction times in even numbers in the regression analysis (cf. Table 1). However, the bivariate correlation with being part of a multiplication table had the opposite direction from regression slopes, suggesting the presence of suppression effects. So at least in the raw correlations, 42 (part of the multiplication table: 6^*^7) would be more even than 46. However, this relation is more tentative than for being a *square* and *divisibility by 4*, because of the reversal of the slope in the multiple regression.

For odd numbers, the interpretation is more difficult, because the prototype and markedness account predict opposing response patterns and our cross-lingual analysis suggest that both may play a role. In line with the outlined markedness strength account, for odd numbers we observed a gradual decrease in response time, starting from prime numbers to numbers that are part of a multiplication table and finally squares. So, 23 (being a prime number) was slower than 27 (being part of the multiplication table (3^*^9), which was slower than a square number (25, but see below). In contrast to those multiplicativity attributes, divisibility by 5 rather followed the prototypicality, as it slowed down responses: (e.g., 45 was slower than 47 or 49, when all other factors (prime, square number) were partialled out)—this is in line with the idea that numbers divisible by 5 are not typical odd numbers and are therefore slower to be categorized as odd. In sum, for odd numbers, we can say that multiplication attributes influence parity decisions strongly and significantly. However, it seems that we are looking at two opposing effects here, markedness strength and prototypicality, which compete with each other. Therefore, a simple order according to RT like for even numbers cannot be provided so easily.

All in all, however, the current data suggest that not all numbers are equally odd or equally even. Several aspects of two-digit numbers, their multiplicativity, their parity congruity, and in some languages their frequency influence parity categorization. Dependent on language, culture, education and predictor, sometimes less prototypical numbers of a category are slower responded to, corroborating the prototypicality account, while in other cases more marked numbers (and in the case of odd numbers, therefore more prototypical numbers) are slower responded to. Which account is most salient for which language and which attribute is an endeavor for future research. However, we wish to acknowledge that methodological constraints like collinearities or having few members of a category might also have influenced the results and produced suppression and interaction effects. This is not a fault of the current study, as we used all two-digit numbers above 19, but instead an inherent attribute of our numerical system. For instance, there are just two even square numbers between 20 and 99, namely 36 and 64 (note that both of them are divisible by four and one of them is also a power of 2). Of course, 2 members in one category is much less than anybody would have liked. Therefore, independent replications of our results are necessary to see how stable the results for a given language will be.^7^

Nevertheless, although not every single multiplicativity predictor (especially for small stimulus groups and high collinearity) may prevail in a replication, the present results quite clearly show that the parity judgments are not all the same. There are some consistent findings that unit and decade magnitude, parity congruity, but also some attributes like being a prime number or being divisible by 4 influence parity decisions in a fairly consistent way across languages. Therefore, we believe it is fair after this study to conclude that not all even/odd numbers are psychologically equally even or odd, respectively. However, we also have to acknowledge that the mechanisms responsible for making numbers more even or odd in a given language or culture need to be better studied and understood in the future.

## Ethics statement

The study was approved by the ethic committee of the Medical Faculty of the University of Tuebingen. It got further approval at other data collection sites (University of York, Department of Psychology and University of Warsaw, Department of Psychology).

## Author contributions

KC, MS, KL, SG, FD, MH, and H-CN designed the study. LH, M-LS collected the data. LH, KC, M-LS, and MS analyzed the data. LH, KC, and MS wrote the manuscript. All authors read and commented on and corrected the manuscript.

### Conflict of interest statement

The authors declare that the research was conducted in the absence of any commercial or financial relationships that could be construed as a potential conflict of interest.

## Footnotes

^1^A group theory in mathematics is about understanding algebraic structures known as groups, which consist of a set of elements and an operation. Here, it provides a background and formal framework for a concept of parity.

^2^On the one hand this result is surprising as number two can be considered as a prototypical even number. On the other hand, it is also a prime number (i.e., it is divisible only by one and by itself). Even more importantly, number 2 is the only even prime number. This property may, at least in some individuals, lead to longer parity decision times to this number.

^3^It was demonstrated several times that phenomena observed in numerical cognition, such as for example the SNARC effect are highly dependent on the task set (see e.g., Dehaene et al., 1993, Exp. 3; Fias et al., 1996, Exp. 1). Thus, to avoid such possible effects, the entire number range was used in the task, and then full decades and tie numbers were excluded *post-hoc*.

^4^We consider this database as the standard in the field of numerical cognition and a better proxy of the real frequency when a cross-lingual design is applied.

^5^Intercorrelations between predictors are considered problematic if they exceed .80. Another value indicating collinearity is the Variance Inflation Factor (VIF), which should not exceed 10 (see e.g., Field et al., 2012; p. 292-293). Some authors recommend even lower acceptable VIF values. To the best of our knowledge, the most conservative threshold is 3. In our case the maximal VIF values were 2.04 and 2.53 for odd and even numbers respectively.

^6^Hines et al. (1996) found a more pronounced odd effect in males when numbers were presented as dot patterns, whereas in females the effect was more pronounced in case of number words. Therefore, in their study the effect and its direction depended on presentation format. They did not use auditory modality, therefore direct replication within our study was not possible. However, to follow-up on their results we checked for gender effects in our data. We did not find a significant Gender × Parity interaction, *F* < 1.00; *p* = 0.842. There was also no Gender × Parity × Language interaction *F* < 0.01; *p* > 0.999.

^7^Note that pairwise matching is probably impossible, because this study suggests that so many different attributes (decade magnitude, unit magnitude, parity congruity, frequency and different multiplicativity attributes) may influence reaction times. These would need to be controlled for pairwise matching, which is probably impossible.
