# Corrigendum: A Mental Odd-Even Continuum Account: Some Numbers May Be “More Odd” Than Others and Some Numbers May Be “More Even” Than Others

**DOI:** 10.3389/fpsyg.2019.01860

**Published:** 2019-08-16

**Authors:** Lia Heubner, Krzysztof Cipora, Mojtaba Soltanlou, Marie-Lene Schlenker, Katarzyna Lipowska, Silke M. Göbel, Frank Domahs, Maciej Haman, Hans-Christoph Nuerk

**Affiliations:** ^1^Department of Psychology, University of Tuebingen, Tuebingen, Germany; ^2^LEAD Graduate School and Research Network, University of Tuebingen, Tuebingen, Germany; ^3^Department of Psychology, University of Warsaw, Warsaw, Poland; ^4^Department of Psychology, University of York, York, United Kingdom; ^5^Institute for German Linguistics, University of Marburg, Marburg, Germany; ^6^Leibniz-Institut für Wissensmedien, Tuebingen, Germany

**Keywords:** parity judgment, markedness, numerical properties, prototypicality, cross-linguistic comparisons

There is an error in the Acknowledgments statement. In the original article, we acknowledge the funder “NCN”. The correct name for the Funder is the “National Science Center (NCN), Poland.”

In addition, the correct funding number for the National Science Center (NCN), Poland is “2014/15/G/HS6/04604.”

The authors apologize for these errors and state that they do not change the scientific conclusions of the article in any way. The original article has been updated.

