# Supplementary material for: A Mental Odd-Even Continuum Account: Some Numbers May Be “More Odd” Than Others and Some Numbers May Be “More Even” Than Others
Source: Front Psychol. 2018 Jun 28;9:1081. doi: 10.3389/fpsyg.2018.01081 (PMC6032609; doi:10.3389/fpsyg.2018.01081)
Supplement: Supplementary file 1 [file Data_Sheet_1.pdf]

Supplementary material to the paper by L. Heubner, K. Cipora, M. Soltanlou, M.-L. Schlenker, K. Lipowska, S.M. Göbel, F. Domahs, M. Haman and H.-C. Nuerk (2018). A mental odd-even continuum account: Some numbers may be "more odd" than others and some numbers may be "more even" than others. *Front. Psychol. 9*:1081. doi: 10.3389/fpsyg.2018.01081

1.1 A: Predictor collinearity

Table A1. Predictor Collinearity – odd numbers

Measure	1	2	3	4	5	6	7	8
1. Decade magnitude	—	-.09	.22	-.06	-.10	-.37*	.03	-.18
2. Unit magnitude		—	-.04	-.00	.01	-.07	.02	.09
3. Parity congruity			—	.05	-.27	-.34*	-.02	-.22
4. Prime number				—	-.29	-.51**	-.46**	-.45**
5. Square					—	.56***	.11	.20
6. Multiplication table						—	.24	.39*
7. Divisibility by 5							—	.51**
8. Frequency log10								—

Note. * $p < .05$, ** $p < .01$, *** $p < .001$

Table A2. Predictor collinearity – even numbers

Measure	1	2	3	4	5	6	7	8
1. Decade magnitude	—	-.14	-.22	-.13	-.33	-.13	-.06	-.40*
2. Unit magnitude		—	.00	.00	-.35	-.25	-.06	-.09
3. Parity congruity			—	.04	-.07	.04	.00	.12
4. Being a square				—	.44*	.46*	.28	.20
5. Multiplication table					—	.44*	.32	.41*
6. Power of 2						—	.28	.20
7. Divisibility by 4							—	.36
8. Frequency log10								—

Note. * $p < .05$

1.2 B: averaged bivariate correlations within participants

Table B1: Averaged bivariate correlations within participants. Positive values show that increasing / possessing given property correlated with longer response times, while negative values that it was associated with shorter response times.

**Odd numbers**	**overall**	**English**	**German**	**Polish**
**Decade magnitude**	0,10	0,13	-0,01	0,18
**Unit magnitude**	0,06	0,02	0,09	0,08
**Parity congruity**	-0,02	-0,03	-0,02	0,00
**Prime number**	0,02	0,01	0,01	0,05
**Square**	-0,01	-0,03	0,00	0,01
**Multiplication table**	-0,04	-0,05	-0,02	-0,04
**Divisibility by 5**	0,05	0,10	0,00	0,05
**Frequency**	0,01	0,06	0,02	-0,05

**Even numbers**	**overall**	**English**	**German**	**Polish**
**Decade magnitude**	0,10	0,06	0,01	0,22
**Unit magnitude**	-0,01	0,01	0,04	-0,08
**Parity congruity**	-0,01	0,00	0,00	-0,04
**Square**	-0,02	-0,02	-0,02	-0,02
**Multiplication table**	-0,02	0,01	0,00	-0,06
**Power of 2**	-0,01	-0,03	0,01	-0,03
**Divisibility by 4**	-0,02	-0,05	-0,01	-0,01
**Frequency**	-0,04	0,03	-0,06	-0,09

1.3 C: SNARC effects

The SNARC effect (Dehaene et al., 1993) was calculated using a method proposed by Fias, Brysbaert, Geypens, and d`Yedewalle (1996). It calculates one single numerical value for each participant, which can be used for further analysis and represents the magnitude of the SNARC effect. First, the difference in reaction times between the both hands ($d_{RT} = RT_{right\ hand} - RT_{left\ hand}$) is calculated for each number for each participant. A left-hand advantage is represented by a positive d_{RT}, a right-hand advantage is represented by a negative d_{RT}. Then the calculated $d_{RT}s$ are regressed on number magnitude. The obtained non-standardized regression slopes can be taken as a measure of the SNARC effect. More negative slopes relate to stronger SNARC effects. To calculate the unit-SNARC effect, the reaction times for each unit number (1, 2, 3, 4, 6, 7, 8, 9) were averaged across decades for each participant separately. In a second step, the same method was used as for the calculation of the overall SNARC effect. To examine the significance of the SNARC effect on sample level, the obtained slopes are tested against zero with a one-sample *t*-test. Following these methods, testing regression slopes for the overall SNARC against 0 did not reveal a significant SNARC effect, $t(106) = -0.21$, $p = .836$. However, testing regression slopes for the unit-SNARC against 0 revealed a significant unit-SNARC effect, $t(106) = -6.15$, $p < .001$.
